# Direct comparison of brain [^18^F]FDG images acquired by SiPM-based and PMT-based PET/CT: phantom and clinical studies

**DOI:** 10.1186/s40658-020-00337-4

**Published:** 2020-11-23

**Authors:** Kei Wagatsuma, Muneyuki Sakata, Kenji Ishibashi, Akira Hirayama, Hirofumi Kawakami, Kenta Miwa, Yukihisa Suzuki, Kenji Ishii

**Affiliations:** 1 Research Team for Neuroimaging, Tokyo Metropolitan Institute of Gerontology, 35-2 Sakae-cho, Itabashi-ku, Tokyo, 173-0015 Japan; 2 GE Healthcare Japan, 4-7-127 Asahigaoka, Hino, 191-8503 Japan; 3 Department of Radiological Sciences, School of Health Science, International University of Health and Welfare, 2600-1 Kitakanemaru, Ohtawara, 324-8501 Japan; 4 Department of Ophthalmology and Visual Science, Tokyo Medical and Dental University, Graduate School, 1-5-45, Yushima, Bunkyo-ku, Tokyo, 113-8510 Japan

**Keywords:** Silicon photomultiplier, Photomultiplier tube, Digital positron emission tomography, Image quality, Standardized uptake value ratio, Statistical image analysis

## Abstract

**Background:**

Silicon photomultiplier-positron emission tomography (SiPM-PET) has better sensitivity, spatial resolution, and timing resolution than photomultiplier tube (PMT)-PET. The present study aimed to clarify the advantages of SiPM-PET in ^18^F-fluoro-2-deoxy-D-glucose ([^18^F]FDG) brain imaging in a head-to-head comparison with PMT-PET in phantom and clinical studies.

**Methods:**

Contrast was calculated from images acquired from a Hoffman 3D brain phantom, and image noise and uniformity were calculated from images acquired from a pool phantom using SiPM- and PMT-PET. Sequential PMT-PET and SiPM-PET [^18^F]FDG images were acquired over a period of 10 min from 22 controls and 10 patients. All images were separately normalized to a standard [^18^F]FDG PET template, then the mean standardized uptake values (SUV_mean_) and *Z*-score were calculated using MIMneuro and CortexID Suite, respectively.

**Results:**

Image contrast, image noise, and uniformity in SiPM-PET changed 19.2, 3.5, and − 40.0% from PMT-PET, respectively. These physical indices of both PET scanners satisfied the criteria for acceptable image quality published by the Japanese Society of Nuclear Medicine of contrast > 55%, CV ≤ 15%, and SD ≤ 0.0249, respectively. Contrast was 70.0% for SiPM-PET without TOF and 59.5% for PMT-PET without TOF. The TOF improved contrast by 3.5% in SiPM-PET. The SUV_mean_ using SiPM-PET was significantly higher than PMT-PET and did not correlate with a time delay. *Z*-scores were also significantly higher in images acquired from SiPM-PET (except for the bilateral posterior cingulate) than PMT-PET because the peak signal that was extracted by the calculation of *Z*-score in CortexID Suite was increased. The hypometabolic area in statistical maps was reduced and localized using SiPM-PET. The trend was independent of whether the images were derived from controls or patients.

**Conclusions:**

The improved spatial resolution and sensitivity of SiPM-PET contributed to better image contrast and uniformity in brain [^18^F]FDG images. The SiPM-PET offers better quality and more accurate quantitation of brain PET images. The SUV_mean_ and *Z*-scores were higher in SiPM-PET than PMT-PET due to improved PVE. [^18^F]FDG images acquired using SiPM-PET will help to improve diagnostic outcomes based on statistical image analysis because SiPM-PET would localize the distribution of glucose metabolism on *Z*-score maps.

**Supplementary Information:**

The online version contains supplementary material available at 10.1186/s40658-020-00337-4.

## Introduction

Positron emission tomography (PET) has become an important imaging technology for evaluating biochemical and physiological functions and pathological abnormalities [1, 2]. Brain imaging with ^18^F-fluoro-2-deoxy-D-glucose ([^18^F]FDG) measures local glucose metabolism as a proxy for neuronal activity and thus is a powerful tool for differentially diagnosing dementia [3, 4].

Silicon photomultipliers (SiPM) developed by Hamamatsu Photonics K.K. have replaced photomultiplier tubes (PMT) in newer PET detector systems [5]. The SiPM is a solid-state photon-counting device comprising 100 to > 10,000 avalanche photodiode pixels in Geiger mode. The desirable features of SiPM comprise good intrinsic timing resolution (< 200 ps, not for clinical use), compact, rugged design, higher gain (similar to that of PMT), and more sensitive photon detection than PMT [5–11]. The scintillator size is a dominant factor in the spatial resolution of PET imaging [12]. The clinical PET scanner using SiPM is likely to improve spatial resolution because it has crystals < 4 × 4 mm [10, 13]. The SiPM gain and photon detection efficiency are temperature-dependent [14, 15]. Therefore, an efficient cooling system is required for SiPM-based PET scanners to maintain performance. The temperature of the Discovery MI (DMI; GE Healthcare, Milwaukee, WI, USA) and the SIGNA PET/MR (GE Healthcare) is maintained at 17–18 °C with a constant flow of coolant [8]. The SiPM-based PET/computed tomography (CT) Discovery MI had a PET axial field-of-view (FOV) of 20 cm, small lutetium-based scintillators (LBS) with a SiPM block design, and a timing resolution of 375 ps [16]. We found that the SiPM-PET had good sensitivity as well as spatial and timing resolution in National Electrical Manufactured Association (NEMA) performance tests. Contrast was better on images acquired from the DMI than the Discovery PET/CT 710 (D710, GE Healthcare) with PMT detectors [17].

The clinical applicability of SiPM-PET/CT has been investigated. Hsu et al. found that SiPM-PET improved the contrast recovery of small lesions [16]. Tiny malignant lesions in a patient with melanoma were detected on [^18^F]FDG images acquired using SiPM-PET/CT and a Bayesian penalized-likelihood reconstruction algorithm [18]. Sonni et al. reported that SiPM technology and the time-of-flight (TOF) algorithm could reduce the duration of whole-body image acquisition without losing image quality [19]. Salvadori et al. compared the quality of brain [^18^F]FDG images between Philips SiPM- and PMT-PET scanners [20] and found better contrast and spatial resolution with less noise, when images were acquired using digital PET in their clinical study. Sluis et al. evaluated the performance of a Siemens SiPM-PET using NEMA tests and visually compared [^18^F]FDG brain images acquired by PMT-PET and SiPM-PET, but did not physically evaluate the quality of [^18^F]FDG brain images [13].

The present study aimed to clarify the advantage of SiPM-PET system in [^18^F]FDG brain imaging in head-to-head comparisons between DMI and D710 in phantom and clinical studies. To our knowledge, this is the first attempt to evaluate SiPM-PET image quality using a Hoffman 3D brain phantom. We also evaluated the results of quantitative analysis and statistical image analysis in a clinical study.

## Materials and methods

### PET/CT systems

#### Discovery MI

The Discovery MI is a combination of an LBS, an SiPM-PET scanner and a 64-slice CT scanner. The LBS includes four blocks of detectors aligned in the axial direction, each comprising 19,584 crystals (3.95 × 5.3 × 25-mm) in a 4 × 9 matrix. The scanner has 36 detector units per ring and 9792 SiPM channels. The DMI enables axial and transaxial FOV of 20 and 70 cm, respectively, with 71 image planes spaced at 2.79-mm intervals. The timing resolution is 375 ps [16]. The spatial resolution according to NEMA NU 2-2012 is 3.91 mm at full width at half maximum (FWHM) [17].

#### Discovery PET/CT 710

The Discovery PET/CT 710 is a combination of LBS with PMT-PET and 64-slice CT scanners. The PET scanner has 13,824 LBS crystals in a 4.2 × 6.3 × 25-mm^3^ block. The D710 enables a 157-mm axial FOV and a 700-mm transaxial FOV with 47 image planes spaced at 3.27-mm intervals. The timing resolution is 500 ps. The spatial resolution according to a NEMA NU 2-2007 is 4.52 mm at FWHM [21].

#### PET reconstruction condition

Data acquired using SiPM-PET and PMT-PET were reconstructed under the following conditions: three-dimensional-ordered subset-expectation maximization (3D-OS-EM) with TOF; 4 iterations; 16 subsets; Gaussian filter, 2.5 mm (FWHM); 128 × 128 matrix size; FOV, 25.6 cm; 2.0 mm/pixel. Images of the Hoffman 3D brain phantom acquired by both PET scanners were also reconstructed without TOF to evaluate changes in image contrast using TOF as the contrast gain. Contrast gain was evaluated as described below.

### Phantom study

#### Data acquisition

Images were acquired for 30 min on different days using SiPM-PET and PMT-PET in list mode from a Hoffman 3D brain phantom (Data Spectrum Corporation, Hillsborough, NC, USA) that mimicked the [^18^F]FDG distribution in the human brain [22] and a pool phantom (Itoi Plastics Co. Ltd., Kobe, Japan), each containing 20 MBq of [^18^F]FDG. Phantom conditions and the scan duration were determined according to the Japanese Society of Nuclear Medicine (JSNM) phantom test procedure [23]. We extracted time frames of 0–420 for the PMT-PET and 0–380 s for SiPM-PET from 30 min of data derived from the two phantoms. The count statistics achieved from the PMT-PET time frame were equivalent to those for [^18^F]FDG clinical brain images at our institution as described below. The time frame of SiPM-PET was determined based on radioactive decay during the scan interval during the second acquisition.

#### Data processing

Image quality was evaluated using physical indices for phantom tests proposed by the JSNM: the ratio of gray-to-white matter contrast (contrast, %) calculated from images of Hoffman phantom and image noise (coefficient of variation, CV [%]) and uniformity (standard deviation, SD) calculated from images of pool phantom [23]. The SD was also calculated from the pool phantom image with a scan duration of 30 min. The contrast, CV, and SD were respectively calculated as described using images acquired from Hoffman and pool phantoms [23]. Contrast gain (%) was calculated as:

$$ \raisebox{1ex}{$\left({Contrast}_{TOF}-{Contrast}_{non- TOF}\right)\times 100$}\!\left/ \!\raisebox{-1ex}{${Contrast}_{non- TOF}$}\right. $$

where *Contrast*_*TOF*_ and *Contrast*_*non-TOF*_ are contrast (%) with and without TOF, respectively.

These physical indices were calculated using PETquactIE ver. 3.0 (Nihon Medi-Physics Co., Ltd., Tokyo, Japan).

### Clinical protocol

#### Data acquisition

The present study proceeded in accordance with the Declaration of Helsinki and was approved by the Ethics Committee at the TMIG (Approval No. 28077). All control individuals and patients provided written informed consent to participate in the present study after physicians provided a detailed explanation of the study at the Research Team for Neuroimaging. The individuals rested comfortably in a quiet, dimly lit room for several minutes, then were placed in the supine position for intravenous [^18^F]FDG injection and uptake. Low-dose CT images for attenuation and scatter correction were acquired before starting PET image acquisition. The first set of PMT-PET images were acquired for 10 min starting from 40 min after [^18^F]FDG administration, and then, the second set of SiPM-PET images was acquired, also for 10 min. The second scan started within 5 min of completing the first scan of 22 controls and 10 patients using the two PET/CT scanners between April 2017 and July 2018. The controls were confirmed as not having degenerative neurological disorders on [^18^F]FDG and brain magnetic resonance images acquired using a Discovery MR750w 3.0T scanner (GE Healthcare). The MR images were acquired under the following conditions: three-dimensional mode (spoiled gradient recalled acquisition in the steady state: repetition time, 7.648 ms; echo time, 3.092 ms; matrix size, 196 × 256 × 256; voxel size, 1.2 × 1.0547 × 1.0547 mm^3^). Table 1 shows the characteristics of the controls. Among the controls, four were healthy volunteers and 18 had visual issues (visual snow, *n* = 12; blepharospasm, *n* = 2; visual disturbance, *n* = 1; photophobia, *n* = 1; Charles Bonnet syndrome, *n* = 1; traffic injury, *n* = 1). Ten patients had suspected degenerative neurological disorders. Table 2 shows the characteristics of patients.

**Table 1:** Characteristics of controls (*n* = 22)

Age (mean ± SD, range)	41.1 ± 18.9, (21 – 75)
Male (n)	11
Height (cm)	166.1 ± 7.5
Weight (kg)	58.5 ± 9.1
Glucose (mg/dL)	101.0 ± 13.7
Injected dose (MBq)	155.8 ± 14.7
Uptake duration (min, PMT-PET/SiPM-PET)	40.1 ± 0.6/55.3 ± 1.2

Data are shown as means ± standard deviation

*PET* Positron emission tomography, *PMT* Photomultiplier tube, *SiPM* Silicon photomultiplier

**Table 2:** Characteristics of patients

Pt.	Clinical diagnosis	Age	Sex	Weight (kg)	Glucose (mg/dL)	Injected dose (MBq)	Uptake duration (min, PMT/SiPM)
1	MCI	72	Male	63.3	96	162.5	40.0/55.6
2	Amnesia	49	Male	73.1	102	151.7	40.0/54.5
3	FTLD	60	Female	63.0	95	149.3	40.0/57.5
4	Juvenile AD	49	Male	89.4	93	168.7	40.0/57.2
5	Juvenile AD	55	Male	65.4	111	172.1	40.0/56.8
6	MCI	70	Female	46.6	90	174.8	40.1/56.6
7	Amnesia, AD	81	Male	55.5	96	165.0	40.0/55.6
8	Dystonia	36	Female	42.9	99	172.2	40.0/54.7
9	Familial AD	55	Female	54.0	96	160.4	40.0/54.5
10	Posterior cortical atrophy	63	Male	61.3	100	140.1	40.0/57.0
Means ± SD	-	59.0 ±13.1	-	61.5 ± 13.3	97.8 ± 5.8	161.7 ±11.4	40.0 ± 0.0/ 56.0 ± 1.2

*AD* Alzheimer’s disease, *FTLD* Frontotemporal lobar degeneration, *MCI* Mild cognitive impairment, *PMT* Photomultiplier tube, *SiPM* Silicon photomultiplier

#### Data processing

We separately normalized [^18^F]FDG images of 22 controls to a standard [^18^F]FDG PET template using MIMneuro (MIM Software Inc. Cleveland, OH, USA). Anatomical volumes of interest (VOI) of MIMneuro were automatically placed on the caudate nucleus; the cerebellum, frontal, occipital, parietal, and temporal lobes; the putamen, thalamus, and whole brain. Mean standardized uptake values (SUV_mean_) were measured using these VOI [24]. Images of the 22 controls and 10 patients were statistically analyzed using CortexID Suite (GE Healthcare) [25–27]. Anatomical VOI of CortexID Suite comprised the lateral and medial frontal, inferior and superior parietal, and lateral and medial temporal lobes, the anterior and posterior cingulate cortex, occipital lobe, sensorimotor, precuneus, primary visual cortex, and cerebellum. The SUV ratio (SUVR) was calculated using the value of the pons as a reference region. *Z*-scores for anatomical VOI-based analyses were calculated from anatomically normalized SUVR images using the formula,

$$ \raisebox{1ex}{$\left({SUVR}_{individual}-{SUVR}_{normal}\right)$}\!\left/ \!\raisebox{-1ex}{${SD}_{normal}$}\right., $$

where *SUVR*_*individual*_ and *SUVR*_*normal*_ are the mean SUVR of the individuals and the normal database of CortexID Suite in the VOI, respectively, and *SD*_*normal*_ is the SD of the SUVR of the normal database of CortexID Suite in the VOI.

#### Data analyses

Data were statistically analyzed using Prism 8 Version 8.4.0 (GraphPad Software Inc., San Diego, CA, USA). The SUV_mean_ of all regions for SiPM- and PMT- PET acquisitions were statistically compared using two-tailed paired Student *t* tests. Spearman rank correlation coefficients were calculated to evaluate relationships among different SUV_mean_ in the whole brain and intervals between acquisitions. *Z*-scores were statistically analyzed for both acquisitions using Wilcoxon matched-pairs signed rank tests. Values with *P* < 0.05 were considered significant.

## Results

### Phantom study

Table 3 shows that the physical indices of both scanners satisfied the JSNM image quality acceptance criteria of contrast > 55%, CV ≤ 15%, and SD ≤ 0.0249. The pool phantom images under the clinical conditions also satisfied the criterion of uniformity. Contrast was higher using SiPM-PET than PMT-PET. Contrast was 70.0% for the SiPM-PET without TOF and 59.5% for the PMT-PET without TOF. The TOF improved the contrast in SiPM-PET by 3.5%.

**Table 3:** Physical indices in JSNM criteria and contrast gain in background of SiPM- and PMT-PET

PET system	Contrast (%)	CV (%)	SD (clinical vs. 30 min)	Contrast gain (%)
PMT-PET	58.5	11.1	0.021/0.018	− 1.7
SiPM-PET	72.4	11.5	0.015/0.009	3.5

JSNM criteria: contrast ratio > 55%, CV < 13%, SD < 0.0249

*CV* Coefficient of variation, *JSNM* Japanese Society of Nuclear Medicine, *PET* Positron emission tomography, *PMT* Photomultiplier tube, *SD* Standard deviation, *SiPM* Silicon photomultiplier

### Clinical study

**Fig. 1:**
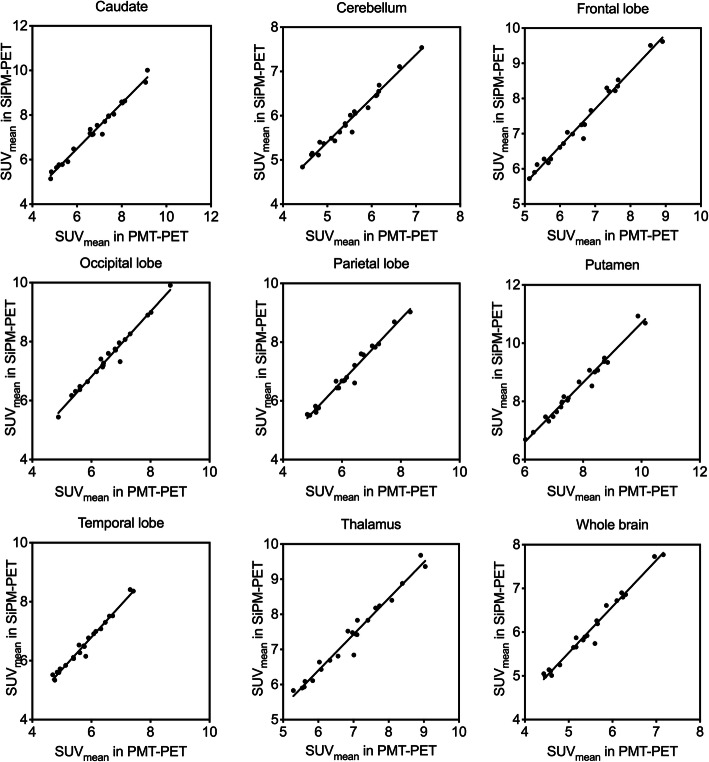
Changes in SUV_mean_ of all regions in images acquired by PMT-PET and SiPM-PET. PET positron emission tomography, PMT photomultiplier tube, SiPM silicon photomultiplier, SUV_mean_ mean standardized uptake value

**Fig. 2:**
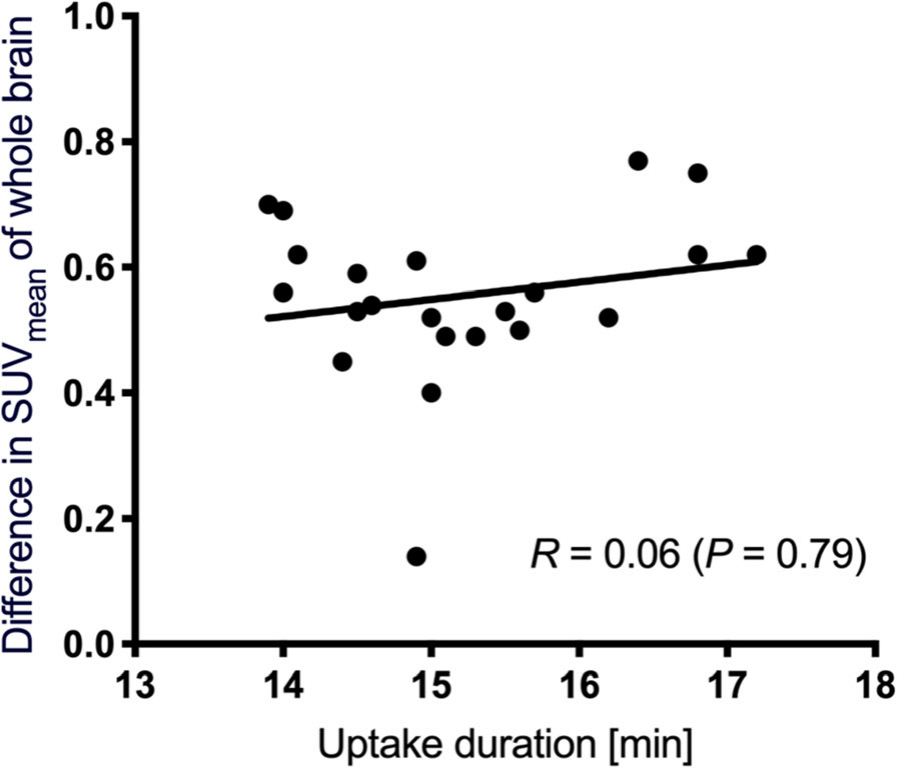
Correlations between changes in SUV_mean_ in whole brain and all regions and time between sequential acquisitions. SUV_mean_ mean standardized uptake value

Figure 1 and Table 4 show changes in SUV_mean_ and mean (± SD) SUV_mean_ across all brain regions of controls, respectively, between acquired using SiPM- and PMT-PET. The SUV_mean_ was significantly higher on SiPM-PET than PMT-PET images in all regions. The mean (± SD) of the interval between sequential acquisitions (PMT-PET followed by SiPM-PET) was 15.2 ± 1.0 min for controls. The second acquisition started about 5 min after the end of the first acquisition. Figure 2 shows correlations between changes of SUV_mean_ in whole brain and time between first and second acquisitions in controls. The *R* of the SUV_mean_ was 0.06 (*P* = 0.79), then the SUV_mean_ was independent of the time.

**Table 4:** Mean standardized uptake values for [^18^F]FDG brain images acquired using SiPM- and PMT-PET (*n* = 22)

Region	PMT-PET	SiPM-PET	Difference
Cerebellum	5.5 ± 0.7	5.9 ± 0.7	7.9%
Brain stem	4.4 ± 0.5	4.6 ± 0.5	5.4%
Caudate	6.8 ± 1.3	7.4 ± 1.3	8.6%
Frontal lobe	6.6 ± 1.0	7.4 ± 1.1	11.0%
Occipital lobe	6.5 ± 0.9	7.5 ± 1.0	14.1%
Parietal lobe	6.2 ± 0.9	6.9 ± 1.0	11.9%
Temporal lobe	5.8 ± 0.8	6.6 ± 0.9	14.2%
Putamen	7.8 ± 1.1	8.5 ± 1.1	9.3%
Thalamus	6.9 ± 1.1	7.4 ± 1.1	7.2%
Whole brain	5.5 ± 0.8	6.1 ± 0.8	11.0%

Data are shown as means ± standard deviation

*PET* Positron emission tomography, *PMT* Photomultiplier tube, *SiPM* Silicon photomultiplier

**Fig. 3:**
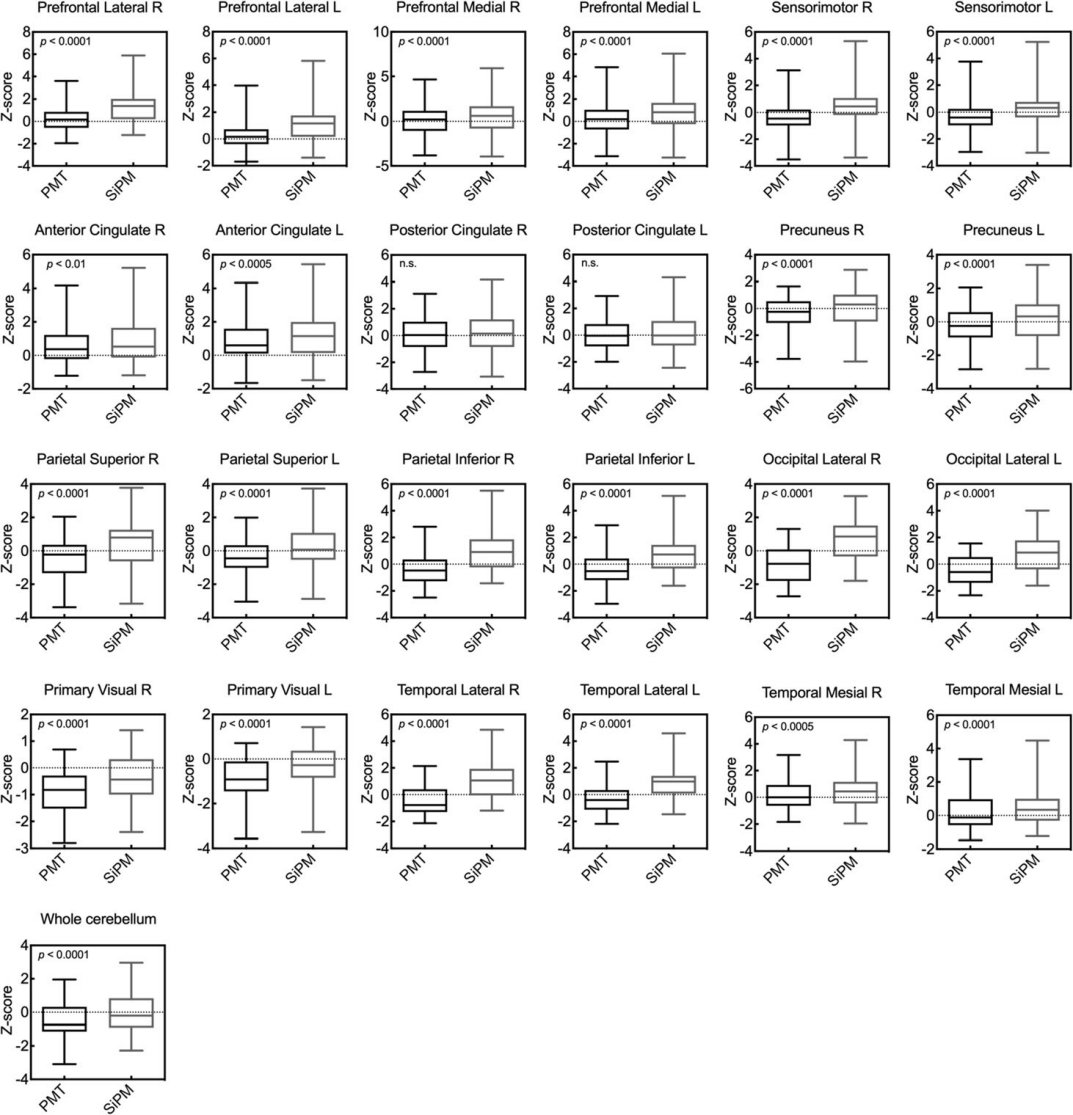
*Z*-scores in all brain regions in SiPM-PET and PMT-PET images. L left, PET positron emission tomography, PMT photomultiplier tube, R right, SiPM silicon photomultiplier

**Fig. 4:**
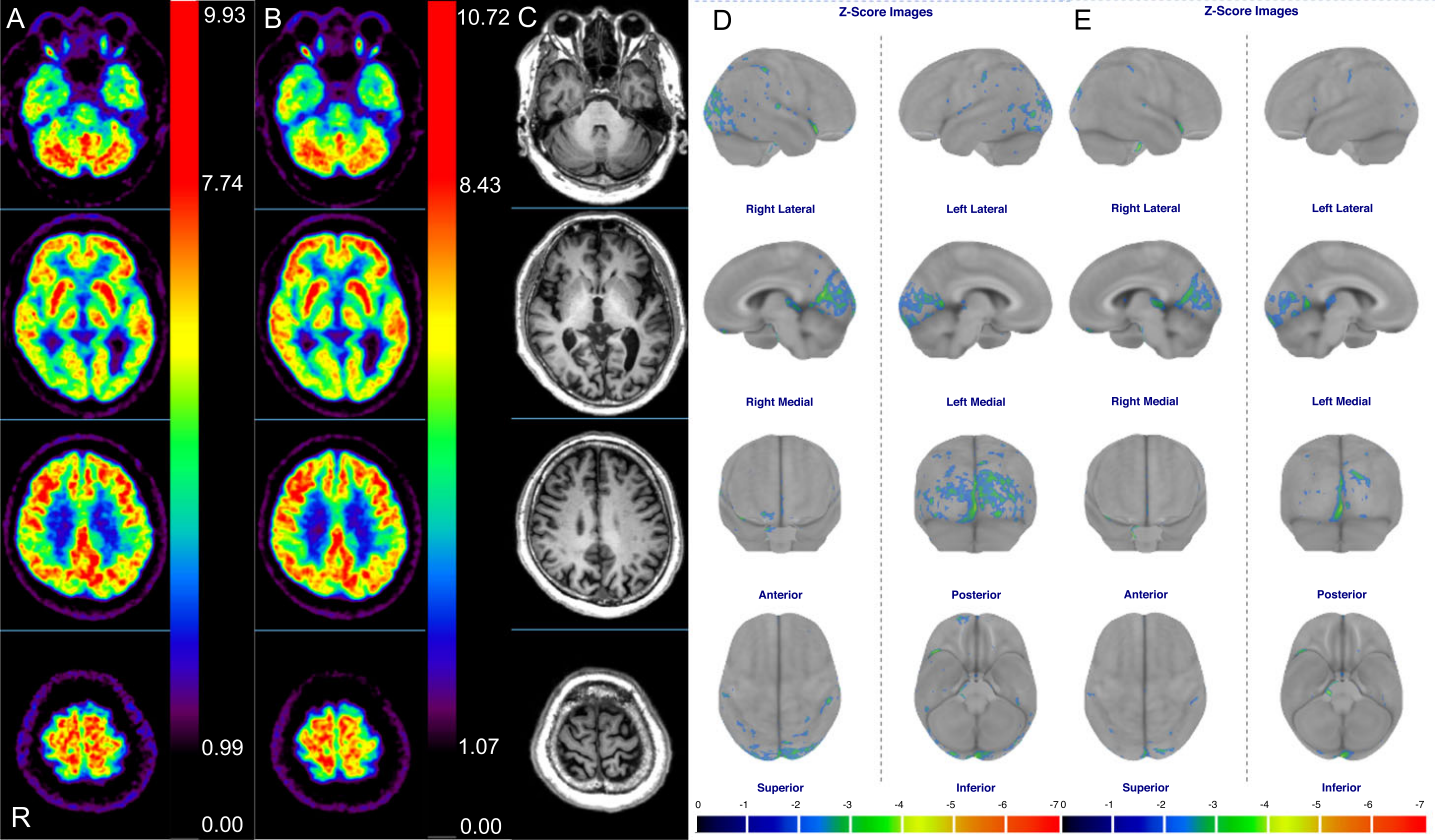
[^18^F]FDG images, MR images, and *Z*-score maps derived from a 71-year-old male. [^18^F]FDG images acquired using PMT-PET (**a**) and SiPM-PET (**b**). MR image of slices of both [^18^F]FDG images (**c**). *Z*-score maps calculated from PMT-PET (**d**) and SiPM-PET (**e**) images. MR magnetic resonance, PET positron emission tomography, PMT photomultiplier tube, SiPM silicon photomultiplier

**Fig. 5:**
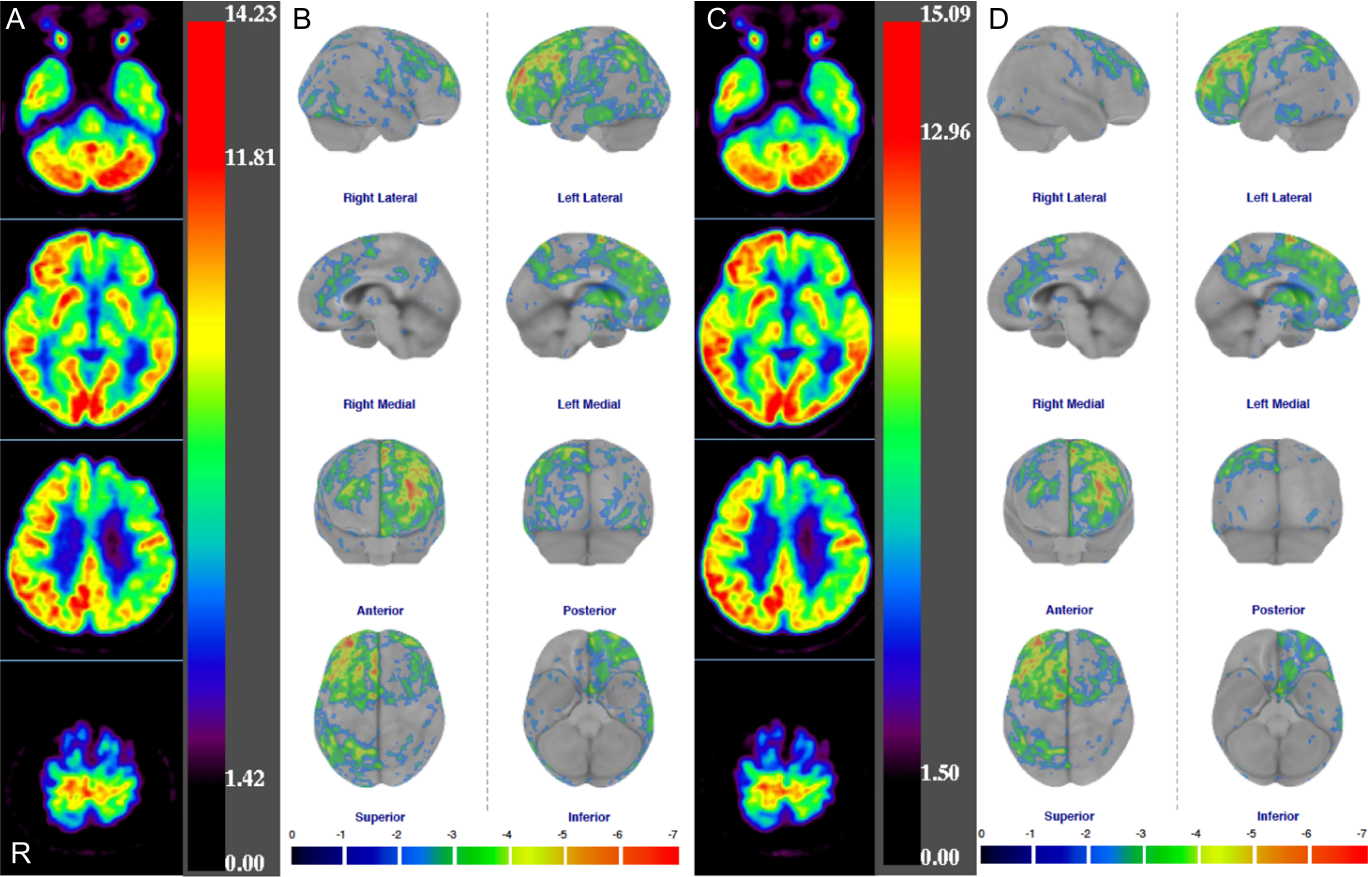
[^18^F]FDG images and *Z*-score maps derived from a 60-year-old female with suspected frontotemporal lobar degeneration. [^18^F]FDG images (**a**) and Z-score maps (**b**) in PMT-PET and [^18^F]FDG images (**c**) and Z-score maps (**d**) in SiPM-PET. PET positron emission tomography, PMT photomultiplier tube, SiPM silicon photomultiplier

Figure 3 shows that the comparisons of *Z*-scores analyzed in all regions (except the bilateral posterior cingulate) using the CotexID Suite were significantly higher in SiPM-PET than in PMT-PET images. Figure 4 shows [^18^F]FDG images, *Z*-score maps, and MR images from a control who was a 71-year-old male with visual hallucinations and Charles Bonnet syndrome (CBS). Glucose metabolization was reduced at the visual association cortex, but hypermetabolism was undetectable on both statistical maps. Figure 5 shows [^18^F]FDG images and *Z*-score maps from a 60-year-old female with suspected frontotemporal lobar degeneration. Glucose metabolism was reduced at the left fontal, temporal, and parietal lobes, the precentral gyrus, striatum, and thalamus on both *Z*-score maps. The hypometabolic area in statistical maps was reduced and localized using SiPM-PET. The trend persisted regardless of whether the images derived from controls or patients (Figs. 4 and 5; Supplements 1 and 2).

## Discussion

We investigated the potential advantages of a new digital SiPM-PET/CT system, DMI, by head-to-head comparisons with a conventional PMT-PET/CT system, D710, in phantom and clinical studies using [^18^F]FDG imaging. Contrast and uniformity were better for the SiPM- than the PMT-PET, and image noise was equivalent between them. The SUV_mean_ was significantly higher in all regions of SiPM-, than PMT-PET images in the clinical study. *Z*-scores were significantly higher in SiPM- than PMT-PET images in all regions except the bilateral posterior cingulate.

The timing and spatial resolution is better for PET systems with SiPM than PMT [5, 6, 8, 9]. The timing resolution is improved due to a bright scintillation crystal with short rise and/or decay times, a low height-width aspect ratio, and a peak emission wavelength that matches the spectral sensitivity of the SiPM [28]. The timing resolution of experimental detector system using SiPM is < 200 ps [7, 10], whereas that of the clinical DMI PET system is 375 ps. The timing resolution of other PET systems developed by Philips and Siemens are 322 and 214 ps, respectively, to use thinner scintillator crystals than our PET system [13, 29]. Commercial clinical SiPM-PET have smaller crystals (< 4.0 mm) than PMT-PET [13, 17, 29]. The SiPM-PET detector improved the ability to detect small lesions, and its features can be summarized as better spatial resolution, timing resolution, and sensitivity than conventional PMT-PET [16–18].

Contrast was better in phantom images acquired using SiPM- than PMT-PET and slightly improved when using SiPM-PET with TOF. The contrast in this study was better than that we previously found by measured radioactivity in the Hoffman phantom using a 1-cm circular region of interest (ROI) [17]. Here, we used a JSNM ROI template that covers regions of gray and white matter, and this suppressed spillover from white matter and increased contrast [23]. The true radioactive distribution ratio in the Hoffman phantom mimicking the cerebral blood flow ratio of gray to white matter is 4. However, the gray to white matter contrast measured on PET images was degenerated by partial volume effects (PVE). The SiPM-PET with a crystal size of < 4 mm decreased PVE and achieved better contrast than PMT-PET. A smaller voxel size also reduces PVE in PET images. We reconstructed Hoffman phantom images using a 256 × 256 matrix with 1-mm pixels and evaluated physical indices (Supplement 3). The contrast was essentially the same between SiPM-PET with a large matrix and clinical conditions (72.8% vs. 72.4%). The ultimate spatial resolution of PET imaging does not fall below half the crystal width [12]. The respective detector widths are 3.95 and 4.2 mm for SiPM-PET and PMT-PET, respectively. We consider that the 128 × 128 matrix with 2-mm pixels is valid for clinical applications. The respective detector widths are 3.95 and 4.2 mm for SiPM- and PMT-PET. The improvement in image contrast (23.8%) using SiPM-PET in the present study was smaller than that in the clinical study of the Philips SiPM-PET because the scintillator crystals were smaller than those in our detector [20]. However, that study did not find a benefit of TOF with an SiPM-PET system for brain PET. Nagaki et al. found that contrast in [^18^F]FDG brain imaging was not improved using the PMT-PET system at a timing resolution of 555 ps [30]. The sensitivity gain using TOF was increased as a function of increasing the object size [31]. The timing resolution was respectively 375 and 544 ps for SiPM- and PMT-PET [16, 21]. These led to spatial localization along a line of response of 5.8 and 7.5 cm, respectively [17]. We considered that the SiPM-PET with TOF would improve image contrast more in brain PET images, but it was slight. Sluis et al. showed clear demarcation of the striatum and thalamus using a Siemens SiPM-PET with a timing resolution of 214 ps [13]. Yoshida et al. developed a brain-dedicated PET system with a timing resolution of 245 ps and generated high-quality images from the Hoffman phantom [32], but did not investigate the benefit of SiPM-PET with TOF using physical indices in brain imaging. We previously generated visually improved [^18^F]FDG brain images using SiPM-PET with TOF [17]. Sluis et al. evaluated the performance of a Siemens SiPM-PET using NEMA tests and visually compared [^18^F]FDG brain images acquired by PMT-PET and SiPM-PET, but did not physically evaluate the quality of [^18^F]FDG brain images [13]. The benefit of TOF with a timing resolution of < 300 ps should be investigated in the future.

Image noise was the same between SiPM-PET and PMT-PET in the present study, although a wider axial FOV contributed to the better sensitivity of DMI compared with the D710 (200 vs. 157 mm) [17]. The statistical noise at the center slice was higher in SiPM-PET than PMT-PET images because the DMI slices were thinner than those of the D710 (2.79 vs. 3.27 mm). The SiPM-PET did not reduce statistical noise under the clinical conditions of 20 MBq of radioactivity and an acquisition duration of 7 min. A trade-off exists between the sensitivity and timing resolution in SiPM-PET. Smaller crystals applied to reduce timing resolution also reduce sensitivity [28, 32]. The sensitivity was equivalent between the DMI with three detector rings (axial FOV, 150 mm) and the D710 (axial FOV, 157 mm) [33, 34]. Thus, we argue that the optimal method to reduce image noise in SiPM-PET is to increase the detector ring to extend the axial FOV.

The phantom images acquired by SiPM-PET had better uniformity. The SD was calculated from the mean radioactivity concentration at the center slice and on the slice ± 40 mm from the center of the pool phantom that corresponded to the cerebellum (− 40 mm) and parietal lobe (+ 40 mm) in the human brain [23]. Uniformity can be estimated as an index of the count stability through the entire axial FOV. Good uniformity means less statistical noise on PET images at the edge of axial FOV. The SiPM-PET could include a whole brain within its PET axial FOV. The statistical noise was suppressed at the bottom of the brain such as the pons and cerebellum which were the reference regions used to calculate the SUVR for [^18^F]FDG [24], amyloid [35, 36], and Tau [37] PET images acquired using SiPM-PET. Therefore, the SUVR calculated by SiPM-PET was expected to be stable.

The clinical study showed that the SUV_mean_ was significantly higher using SiPM-PET than PMT-PET and did not correlate with the delay after injection [38]. The superior spatial resolution by SiPM-PET not only improved image contrast but also increased the SUV_mean_ in the cortex [39]. The higher *Z*-scores determined using SiPM-PET was affected by a higher SUV_mean_. CortexID Suite uses three-dimensional stereotactic surface projections (3D-SSP) for statistical image analysis [25]. The SiPM-PET raised the peak signal on the cortex that was used to analyze the 3D-SSP because small scintillator crystals in the SiPM-PET reduced PVE in the signal of gray matter [16]. Salvadori et al. also found better recovery coefficients in SiPM- than PMT-PET even at the same pixel size (20). On the other hand, the Japanese Alzheimer Disease Neuroimaging Initiative study found higher [^18^F]FDG distribution in late (55–60 min) than in early (30–35 min) scans of the frontal and parietal lobes [40]. We also found a higher SUV_mean_ in the second compared with the first scan of the frontal and parietal lobes. However, the difference in SUV_mean_ in these lobes did not correlate with elapsed time from injection (data not shown). We argue that the SiPM-PET contributes to improving signal loss resulting from PVE in brain PET imaging.

Significant hypometabolism was detected in the primary visual and visual association cortices on *Z*-score maps (Fig. 5d, e). Charles Bonnet syndrome appeared as hypometabolism in the primary visual cortex and hypermetabolism in the visual association cortex on [^18^F]FDG PET images [41]. Hypermetabolism was undetectable in the visual association cortex because this person might have had fewer or milder symptoms of CBS than visual hallucinations. The statistical hypometabolic areas were reduced and localized by SiPM-PET in *Z*-score maps of controls and patients. The SiPM-PET improved the PVE and expressed more accurate metabolic distribution than PMT-PET. Misdiagnosis in dementia corrected using 3D-SSP has significantly enhanced the diagnostic confidence of nuclear medicine physicians [42]. False positive findings in the CortexID Suite can be reduced using SiPM-PET. Thus, [^18^F]FDG images acquired using SiPM-PET will help to improve diagnostic outcomes based on statistical image analysis.

The present study has some limitations. We initially investigated a few patients with neurological disorders. The tendency for *Z*-scores and hypometabolic areas to differ was equivalent between SiPM- and PMT-PET images of patients with neurological disorders and controls. The detectability and diagnostic performance of patients with neurological disorders should be assessed in a larger population of [^18^F]FDG brain images using SiPM-PET. Secondly, two acquisitions using SiPM- and PMT-PET proceeded in a specific order. Sequential acquisition in inverse order should be applied to determine actual changes in SUV, although the SUV did not correlate with the amount of elapsed time from injection in the present study. The normal [^18^F]FDG database in the CortexID Suite does not include image data acquired by more recent PET scanners such as SiPM-PET. The diagnostic performance of the CortexID Suite would certainly improve with an updated normal database.

## Conclusions

The improved spatial resolution and sensitivity of SiPM-PET contributed to better image contrast and uniformity in brain [^18^F]FDG images. The SiPM-PET offers better quality and more accurate quantitation of brain PET images. The SUV_mean_ and *Z*-score were higher in SiPM- than PMT-PET due to improved PVE. The [^18^F]FDG images acquired using SiPM-PET will help to improve diagnostic outcomes based on statistical image analysis because the SiPM-PET would localize the distribution of glucose metabolism on *Z*-score maps.

## Supplementary Information

**Additional file 1:** **Supplement 1.** Z-score maps of bilateral and bimedial images in 22 controls. The color scale is -7.0 to 0.0 of Z-score. PET, positron emission tomography. PMT, photomultiplier tubes; SiPM, silicon photomultiplier.

**Additional file 2:** **Supplement 2.** Z-score maps of bilateral and bimedial images in 10 patients. The color scale is -7.0 to 0.0 of Z-score.

**Additional file 3:** **Supplement 3.** Physical indices calculated from two matrix size in PMT-PET and SiPM-PET.

## Data Availability

All data generated and analyzed during this study are included in this published article.

## Abbreviations

[^18^F]FDG: ^18^F-fluoro-2-deoxy-D-glucose
3D-OS-EM: Three dimensional-ordered subset-expectation maximization
3D-SSP: Three-dimensional stereotactic surface projections
AD: Alzheimer’s disease
CBS: Charles Bonnet syndrome
CT: Computed tomography
CV: Coefficient of variation
D710: Discovery PET/CT 710
DMI: Discovery MI
FOV: Field-of-view
FTLD: Frontotemporal lobar degeneration
FWHM: Full width at half maximum
Hoffman phantom: Hoffman 3D brain phantom
JSNM: Japanese Society of Nuclear Medicine
L: Left
LBS: Lutetium-based scintillators
MCI: Mild cognitive impairment
NEMA: National electrical manufactured association
No.: Number
PET: Positron emission tomography
PMT: Photomultiplier tubes
R: Right
ROI: Region of interest
SD: Standard deviation
SiPM: Silicon photomultiplier
SUV_mean_: Mean standardized uptake value
SUVR: SUV ratio
TMIG: Tokyo metropolitan institute of gerontology
TOF: Time-of-flight
VOI: Volume of interest

## References

[1] Wahl RL, Jacene H, Kasamon Y, Lodge MA (2009). From RECIST to PERCIST: evolving Considerations for PET response criteria in solid tumors. J Nucl Med.

[2] Herholz K, Westwood S, Haense C, Dunn G (2011). Evaluation of a calibrated (18)F-FDG PET score as a biomarker for progression in Alzheimer disease and mild cognitive impairment. J Nucl Med.

[3] Foster NL, Heidebrink JL, Clark CM, Jagust WJ, Arnold SE, Barbas NR (2007). FDG-PET improves accuracy in distinguishing frontotemporal dementia and Alzheimer’s disease. Brain.

[4] Rabinovici GD, Rosen HJ, Alkalay A, Kornak J, Furst AJ, Agarwal N (2011). Amyloid vs FDG-PET in the differential diagnosis of AD and FTLD. Neurology.

[5] Kataoka J, Kishimoto A, Fujita T, Nishiyama T, Kurei Y, Tsujikawa T (2015). Recent progress of MPPC-based scintillation detectors in high precision X-ray and gamma-ray imaging. Nucl Instrum Methods Phys Res A Accelerators Spectrometers Detectors Assoc Equip.

[6] David S, Georgiou M, Fysikopoulos E, Loudos G (2015). Evaluation of a SiPM array coupled to a Gd_3_Al_2_Ga_3_O_12_:Ce (GAGG:Ce) discrete scintillator. Phys Med.

[7] Huizenga J, Seifert S, Schreuder F, van Dam HT, Dendooven P, Löhner H (2012). A fast preamplifier concept for SiPM-based time-of-flight PET detectors. Nucl Instrum Methods Phys Res A.

[8] Levin CS, Maramraju SH, Khalighi MM, Deller TW, Delso G, Jansen F (2016). Design features and mutual compatibility studies of the time-of-flight PET capable GE SIGNA PET/MR System. IEEE Trans Med Imaging.

[9] Peng H, Levin LC (2010). Recent developments in PET instrumentation. Curr Pharm Biotechnol.

[10] Schaart DR, Seifert S, Vinke R, van Dam HT, Dendooven P, Löhner H (2010). LaBr_3_: Ce and SiPMs for time-of-flight PET: achieving 100 ps coincidence resolving time. Phys Med Biol.

[11] Slomka PJ, Pan T, Germano G (2016). Recent advances and future progress in PET instrumentation. Semin Nucl Med..

[12] Moses WW. Fundamental limits of spatial resolution in PET. Nucl Instrum Methods Phys Res A. 2011;648(Supplement 1):S236-40. 10.1016/j.nima.2010.11.092 PMC3144741 21804677

[13] van Sluis J, Boellaard R, Somasundaram A, van Snick P, Borra R, Dierckx R (2020). Image quality and semiquantitative measurements on the biograph vision PET/CT system: initial experiences and comparison with the biograph mCT. J Nucl Med..

[14] Buzhan P, Dolgoshein B, Ilyin A, Kantserov V, Kaplin V, Karakash A, et al. An advanced study of silicon photomultiplier. Adv Technol Part Phys. 2002:717–28.

[15] Buzhan P, Dolgoshein B, Filatov L, Ilyin A, Kantzerov V, Kaplin V (2003). Silicon photomultiplier and its possible applications. Nuclear Instruments and Methods in Physics Research Section A..

[16] Hsu DF, Ilan E, Peterson WT, Uribe J, Lubberink M, Levin CS (2017). Studies of a next-generation silicon-photomultiplier-based time-of-flight PET/CT system. J Nucl Med.

[17] Wagatsuma K, Miwa K, Sakata M, Oda K, Ono H, Kameyama M (2017). Comparison between new-generation SiPM-based and conventional PMT-based TOF-PET/CT. Phys Med.

[18] Aljared A, Alharbi AA, Huellner MW (2018). BSREM reconstruction for improved detection of in-transit metastases with digital FDG-PET/CT in patients with malignant melanoma. Clin Nucl Med.

[19] Sonni I, Baratto L, Park S, Hatami N, Srinivas S, Davidzon G (2018). Initial experience with a SiPM-based PET/CT scanner: influence of acquisition time on image quality. EJNMMI Phys.

[20] Salvadori J, Imbert L, Perrin M, Karcher G, Lamiral Z, Marie PY (2019). Head-to-head comparison of image quality between brain ^18^F-FDG images recorded with a fully digital versus a last-generation analog PET camera. EJNMMI Res.

[21] Bettinardi V, Presotto L, Rapisarda E, Picchio M, Gianolli L, Gilardi MC (2011). Physical performance of the new hybrid PETCT Discovery-690. Med Phys.

[22] Hoffman EJ, Cutler PD, Guerrero TM, Digby WM, Mazziotta JC (1991). Assessment of accuracy of PET utilizing a 3-D phantom to simulate the activity distribution of [^18^F]fluorodeoxyglucose uptake in the human brain. J Cereb Blood Flow Metab.

[23] Akamatsu G, Ikari Y, Nishio T, Nishida H, Ohnishi A, Aita K (2016). Optimization of image reconstruction conditions with phantoms for brain FDG and amyloid PET imaging. Ann Nucl Med.

[24] Partovi S, Yuh R, Pirozzi S, Lu Z, Couturier S, Grosse U (2017). Diagnostic performance of an automated analysis software for the diagnosis of Alzheimer’s dementia with ^18^F FDG PET. Am J Nucl Med Mol Imaging.

[25] Minoshima S, Frey KA, Koeppe RA, Foster NL, Kuhl DE (1995). A diagnostic approach in Alzheimer’s disease using three-dimensional stereotactic surface projections of fluorine-18-FDG PET. J Nucl Med.

[26] Josephs KA, Duffy JR, Strand EA, Machulda MM, Senjem ML, Master AV (2012). Characterizing a neurodegenerative syndrome: primary progressive apraxia of speech. Brain.

[27] Probasco JC, Solnes L, Nalluri A, Cohen J, Jones KM, Zan E (2017). Abnormal brain metabolism on FDG-PET/CT is a common early finding in autoimmune encephalitis. Neurol Neuroimmunol Neuroinflamm..

[28] Yeom JY, Vinke R, Levin CS (2013). Optimizing timing performance of silicon photomultiplier-based scintillation detectors. Phys Med Biol.

[29] Zhang J, Maniawski P, Knopp MV (2018). Performance evaluation of the next generation solid-state digital photon counting PET/CT system. EJNMMI Res.

[30] Nagaki A, Onoguchi M, Matsutomo N (2014). Clinical validation of high-resolution image reconstruction algorithms in brain ^18^F-FDG-PET: effect of incorporating Gaussian filter, point spread function, and time-of-flight. Nucl Med Commun.

[31] Surti S (2015). Update on time-of-flight PET imaging. J Nucl Med.

[32] Yoshida E, Tashima H, Akamatsu G, Iwao Y, Takahashi M, Yamashita T, et al. 245 ps-TOF brain-dedicated PET prototype with a hemispherical detector arrangement. Phys Med Biol. 2020; [Epub ahead of print]. 10.1088/1361-6560/ab8c91 32325448

[33] Levin C, Peterson W, Ross S, Stearns C, Uribe J (2016). PET performance as a function of axial field of view for a new silicon photomultiplier-based whole body TOF PET/CT system. J Nucl Med.

[34] Pan T, Einstein SA, Kappadath SC, Grogg KS, Gomez CL, Alessio AM (2019). Performance evaluation of the 5-Ring GE Discovery MI PET/CT system using the national electrical manufacturers association NU 2-2012 Standard. Med Phys..

[35] Joshi AD, Pontecorvo MJ, Lu M, Skovronsky DM, Mintun MA, Devous MD (2015). A semiautomated method for quantification of F 18 Florbetapir PET images. J Nucl Med.

[36] Nelissen N, Van Laere K, Thurfjell L, Owenius R, Vandenbulcke M, Koole M (2009). Phase 1 study of the Pittsburgh compound B derivative ^18^F-flutemetamol in healthy volunteers and patients with probable Alzheimer disease. J Nucl Med.

[37] Barret O, Alagille D, Sanabria S, Comley RA, Weimer RM, Borroni E (2017). Kinetic Modeling of the Tau PET Tracer ^18^F-AV-1451 in human healthy volunteers and Alzheimer disease subjects. J Nucl Med.

[38] Baratto L, Park SY, Hatami N, Davidzon G, Srinivas S, Gambhir SS (2017). ^18^F-FDG silicon photomultiplier PET/CT: a pilot study comparing semi-quantitative measurements with standard PET/CT. PLoS One.

[39] Lindström E, Sundin A, Trampal C, Lindsjö L, Ilan E, Danfors T (2018). Evaluation of penalized-likelihood estimation reconstruction on a digital time-of-flight PET/CT scanner for ^18^F-FDG whole-body examinations. J Nucl Med.

[40] Takahashi R, Ishii K, Senda M, Ito K, Ishii K, Kato T (2013). Equal sensitivity of early and late scans after injection of FDG for the detection of Alzheimer pattern: an analysis of 3D PET data from J-ADNI, a multi-center study. Ann Nucl Med.

[41] Garde N, Skripuletz T, Pul R, Berding G, Weissenborn K, Trebst C (2011). Visual hallucinations in Charles Bonnet syndrome can be seen in fluorodeoxyglucose-PET. J Neuropsychiatry Clin Neurosci..

[42] Kim J, Cho SG, Song M, Kang SR, Kwon SY, Choi KH (2016). Usefulness of 3-dimensional stereotactic surface projection FDG PET images for the diagnosis of dementia. Medicine (Baltimore)..

