# Supplementary figures and images for: Direct comparison of brain [18F]FDG images acquired by SiPM-based and PMT-based PET/CT: phantom and clinical studies
Source: EJNMMI Phys. 2020 Nov 23;7:70. doi: 10.1186/s40658-020-00337-4 (PMC7683764; doi:10.1186/s40658-020-00337-4)

PMT-PET

SiPM-PET

PMT-PET

SiPM-PET

PMT-PET

SiPM-PET

PMT-PET

SiPM-PET

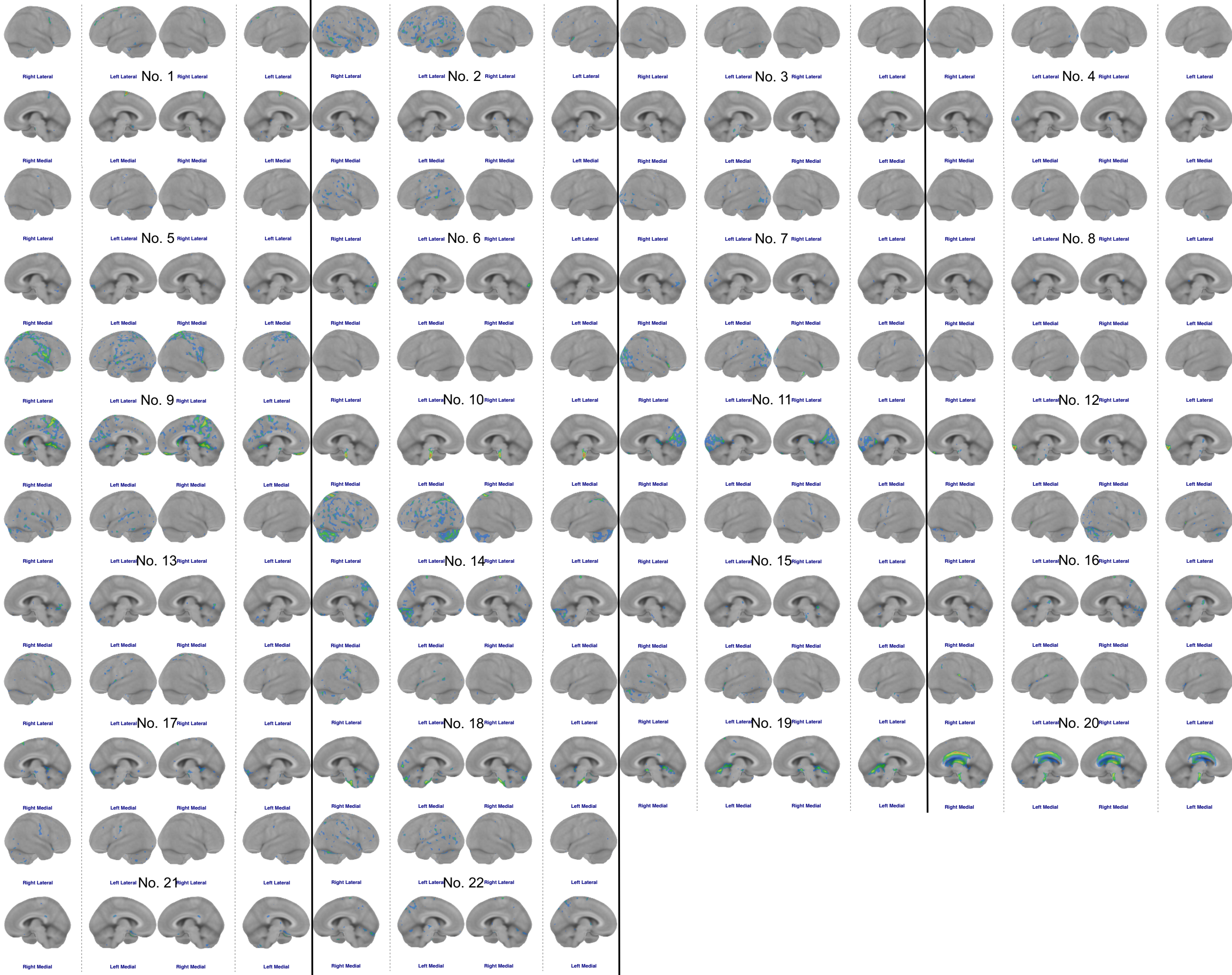

Supplement: Supplementary file 1 — Additional file 1: Supplement 1. Z-score maps of bilateral and bimedial images in 22 controls. The color scale is -7.0 to 0.0 of Z-score. PET, positron emission tomography. PMT, photomultiplier tubes; SiPM, silicon photomultiplier. [file 40658_2020_337_MOESM1_ESM.pdf]

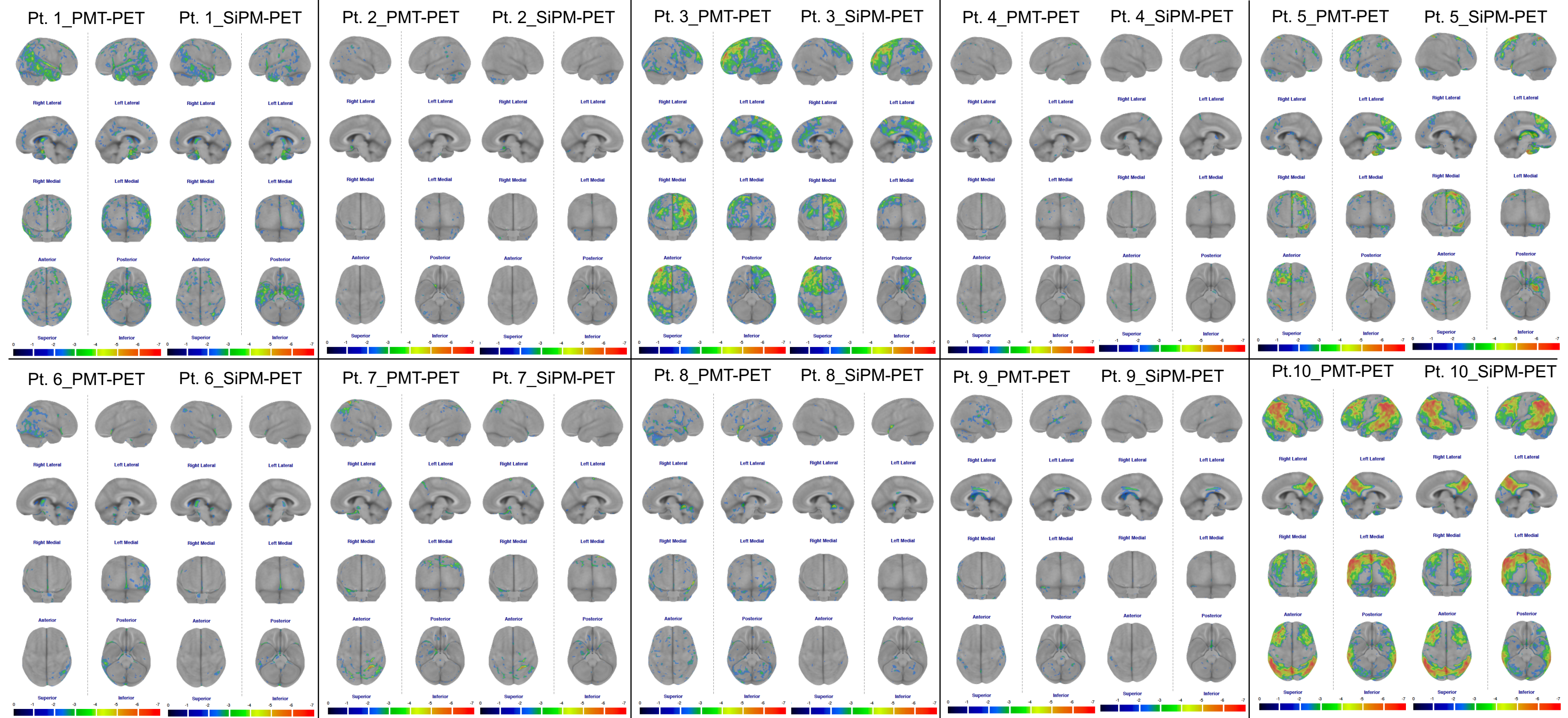

Supplement: Supplementary file 2 — Additional file 2: Supplement 2. Z-score maps of bilateral and bimedial images in 10 patients. The color scale is -7.0 to 0.0 of Z-score. [file 40658_2020_337_MOESM2_ESM.pdf]
