# Supplementary material for: Direct comparison of brain [18F]FDG images acquired by SiPM-based and PMT-based PET/CT: phantom and clinical studies
Source: EJNMMI Phys. 2020 Nov 23;7:70. doi: 10.1186/s40658-020-00337-4 (PMC7683764; doi:10.1186/s40658-020-00337-4)
Supplement: Supplementary file 3 — Additional file 3: Supplement 3. Physical indices calculated from two matrix size in PMT-PET and SiPM-PET. [file 40658_2020_337_MOESM3_ESM.docx]

**Supplement 3.** Physical indices calculated from two matrix size in PMT-PET and SiPM-PET

| PET system | Matrix size | Contrast (%) | CV (%) | SD (Clinical vs. 30 min) |
| --- | --- | --- | --- | --- |
| PMT-PET | 128 × 128 | 58.5 | 11.1 | 0.021/0.018 |
| PMT-PET | 256 × 256 | 58.5 | 10.5 | 0.021/0.018 |
| SiPM-PET | 128 × 128 | 72.4 | 11.5 | 0.015/0.009 |
| SiPM-PET | 256 × 256 | 72.8 | 10.9 | 0.016/0.009 |

CV, coefficient of variation; PET, positron emission tomography; PMT, photomultiplier tube; SD, standard deviation; SiPM, silicon photomultiplier.
